# Urethral Stricture

**Published:** 1878-11

**Authors:** 


*Urethral Stricture.* By Thos. R. Brown, M. D.

The author of this pamphlet is Professor of Clinical and Operative Surgery, and Diseases of the Genito-Urinary Organs, in the College of Physicians and Surgeons, of Baltimore, and full justice is done to the subject, rendering the paper an able essay.

